# LMA Supreme for neonatal resuscitation: study protocol for a randomized controlled trial

**DOI:** 10.1186/1745-6215-15-285

**Published:** 2014-07-15

**Authors:** Daniele Trevisanuto, Francesco Cavallin, Veronica Mardegan, Nguyen Ngoc Loi, Nguyen Viet Tien, Tran Dieu Linh, Tran Dinh Chien, Nicoletta Doglioni, Lino Chiandetti, Luciano Moccia

**Affiliations:** 1 Department of Women and Children Health, University of Padua, Via Giustiniani, 3, Azienda Ospedaliera di Padova, Padova 35128, Italy; 2 Amici della Neonatologia Trentina, Trento, Italy; 3 Independent statistician, Padova, Italy; 4 Department of Neonatal Intensive Care, National Hospital of Obstetrics and Gynecology, Ha Noi, Vietnam; 5 Breath of Life Program - East Meets West Foundation, Oakland, CA, USA

**Keywords:** Laryngeal mask, Resuscitation, Positive pressure ventilation, Newborn infant

## Abstract

**Background:**

The most important action in the resuscitation of a newborn in the delivery room is to establish effective assisted ventilation. The face mask and endotracheal tube are the devices used to achieve this goal. Laryngeal mask airways that fit over the laryngeal inlet have been shown to be effective for ventilating newborns at birth and should be considered as an alternative to facemask ventilation or endotracheal intubation among newborns weighing >2,000 g or delivered ≥34 weeks’ gestation. A recent systematic review and meta-analysis of supraglottic airways in neonatal resuscitation reported the results of four randomized controlled trials (RCTs) stating that fewer infants in the group using laryngeal mask airways required endotracheal intubation (1.5%) compared to the group using face masks (12.0%). However, there were methodological concerns over all the RCTs including the fact that the majority of the operators in the trials were anesthesiologists.

Our hypothesis is based on the assumption that ventilating newborns needing positive pressure ventilation with a laryngeal mask airway will be more effective than ventilating with a face mask in a setting where neonatal resuscitation is performed by midwives, nurses, and pediatricians. The primary aim of this study will be to assess the effectiveness of the laryngeal mask airway over the face mask in preventing the need for endotracheal intubation.

**Methods/design:**

This will be an open, prospective, randomized, single center, clinical trial. In this study, 142 newborns weighing >1,500 g or delivered ≥34 weeks gestation needing positive pressure ventilation at birth will be randomized to be ventilated with a laryngeal mask airway (LMA Supreme^TM^, LMA Company, UK - intervention group) or with a face mask (control group). Primary outcome: Proportion of newborns needing endotracheal intubation. Secondary outcomes: Apgar score at 5 minutes, time to first breath, onset of the first cry, duration of resuscitation, death or moderate to severe hypoxic-ischemic encephalopathy within 7 days of life.

**Trial registration:**

ClinicalTrials.gov identifier: NCT01963936 (October 11, 2013).

## Background

The ability to maintain a patent airway and provide effective positive pressure ventilation (PPV) is the main objective of neonatal resuscitation [1,2]. This is currently achieved with the use of a face mask (FM) or an endotracheal tube (ETT). Both these devices have major limitations from a strictly anatomical point of view and require adequate operator skills. In certain situations, both FM ventilation and ETT intubation may prove difficult to establish an upper airway [3,4].

In 1981, Archie Brain designed the laryngeal mask airway (LMA) with the aim of producing an airway device that would be more practical than the FM and less invasive than the ETT [5].

In adults, the LMA is routinely used during anesthesiology procedures.

The potential advantages of the LMA over the FM include an easier insertion technique, less manipulation of the patient’s head, neck, and jaw, a better airtight seal after positioning, and more effective PPV [6]. Ease of positioning and reduced invasiveness are the reported advantages of the LMA when compared to the tracheal tube [6].

A recent study including 11,910 anesthesia pediatric cases showed that only 102 cases (0.86%) experienced LMA failure. Common presenting features of LMA failures included leak (25%), obstruction (48%), and patient intolerance such as intractable coughing/bucking (11%) [7].

In the setting of the neonatal resuscitation, previous observational studies showed that, when used by teams with expertise (that is, anesthesiologists), the LMA provided effective PPV in most of the treated patients (range 95 to 99%) [8-10]. A 2005 Cochrane review concluded that there was no evidence to evaluate the safety or efficacy for the use of LMA versus FM ventilation in the resuscitation of newborn infants [11]. It suggested that a well-designed randomized controlled trial (RCT) comparing these two airway adjuncts was warranted [11]. In 2013, a systematic review and meta-analysis of supraglottic airways in neonatal resuscitation reported the results of four RCTs, stating that fewer infants in the LMA group required endotracheal intubation (1.5%) compared to the FM group (12.0%) [12]. There were however, methodological concerns over all the RCTs including the fact that the majority of the operators in the trials were anesthesiologists (who are less likely in most clinical settings to be present at neonatal resuscitation).

The LMA is used more often by anesthesiologists rather than pediatricians, nurses, and midwives. It is thus essential to demonstrate its effectiveness by those who will be more commonly involved in neonatal resuscitation in most clinical settings. Furthermore, it is important to thoroughly record any side effects, as in a case series of a comparison of LMA over FM in infants in the operating theatre, significantly more side effects were reported using the LMA over the FM [13,14].

Although previous studies showed that the LMA was effective also in preterm infants weighing less than 2,000 g [9,10], the last version of the International Guidelines for Neonatal Resuscitation state that “a LMA should be considered during resuscitation if FM ventilation is unsuccessful and tracheal intubation is unsuccessful or not feasible. The LMA may be considered as an alternative to a FM for PPV among newborns weighing >2,000 g or delivered ≥34 weeks’ gestation” [1,2].

Despite this recommendation, it has not yet been shown in a well-conducted RCT whether or not LMA is more effective than FM in resuscitation of newborn infants.

## Methods/design

### Aim

The primary aim of this study will be to assess the effectiveness of LMA over FM ventilation in preventing the need for endotracheal intubation at birth.

### Study design

This is a single center, prospective, unblinded, randomized clinical trial of LMA ventilation versus FM ventilation in infants weighing >1,500 g or delivered ≥34 weeks’ gestation.

### Inclusion criteria

Inborn infants satisfying the following inclusion criteria will be eligible to participate in the study:

1. gestational age ≥34 weeks (and)

2. expected birth weight >1,500 g [9,10] (and)

3. need for PPV at birth; the need for PPV will be determined by the presence of apnoea or gasping, or heart rate <100 beats per minute (bpm) after initial resuscitation measures (providing warmth, positioning, clearing the airway, drying and stimulation) over the first 30 seconds [1,2] (and)

4. parental consent; a written informed consent will be obtained by a member of the neonatal team involved in the study from a parent or guardian before delivery.

### Exclusion criteria

1. Lethal anomalies.

2. Hydrops.

3. Major malformations of the respiratory system.

4. Congenital heart disease.

5. Stillbirths; a stillbirth will be diagnosed when a heart rate is never established.

### Primary outcome measure

The primary outcome of this study will be the proportion of newborns needing endotracheal intubation.

### Secondary outcome measures

1. Apgar score at 5 minutes.

2. Time to first breath, defined as the first respiratory effort.

3. Time to first cry, defined as the first audible cry spontaneously emitted by the infant.

4. Death or moderate to severe hypoxic-ischemic encephalopathy (HIE) within 7 days of life, according to a modification of Sarnat and Sarnat [15,16]. According to this classification, HIE grade I (mild) includes irritability, hyperalertness, mild hypotonia, and poor sucking; grade II (moderate) includes lethargy, seizures, marked abnormalities of tone, and requirement of tube feeding; grade III (severe) includes coma, prolonged seizures, severe hypotonia, and failure to maintain spontaneous respiration.

5. Complications secondary to the procedure.

6. Admission to NICU/normal nursery.

### Other collected data

The following data will be collected during resuscitation: (1) Apgar score at 1 min after birth; (2) LMA insertion time, the rate of successful insertion at the first attempt, and the number of attempts required to insert the LMA successfully; (3) adverse effects during resuscitation.

### Generalizability

The findings of this study will be important for other units/settings in high as well low resource countries where neonatal resuscitation is more often performed by pediatricians, midwives, or nurses. Based on the results of the present study, we could speculate whether a short-term educational program on the LMA use will be effective in the clinical practice; furthermore, we will be able to understand whether personnel involved in neonatal resuscitation should be trained to start resuscitation with an FM or with an LMA. Finally, potential complications and side effects due to the LMA will be strictly monitored and collected.

### Sample size

It is estimated that 5% of newborns receive resuscitation with PPV [1,2]. Therefore, in C hospital, Hanoi, Vietnam with more than 20,000 deliveries per year, approximately 1,000 patients will require resuscitation. Of these, about 90% (900 neonates) are newborns weighing >1,500 g or delivered ≥34 weeks gestation.

The sample size was based on a previous study in which the success rate of LMA and FM were 99% and 84%, respectively [17]. To obtain a 90% power at a 0.05 level of significance (one-sided), at least 58 subjects per group need to be enrolled. The number of patients was increased by 20% for each group considering the possibility of dropouts, leading to a final sample of 142 subjects.

### Recruitment

Written and oral information will, whenever possible, be offered to parents prior to the birth of their child if the infant is likely to be eligible. Informed written consent will be signed by a parent or guardian. A senior investigator will be available at all times to discuss concerns raised by parents or clinicians during the course of the trial.

### Randomization

Eligible infants will be assigned to the LMA or the FM group in a 1:1 ratio according to a computer-generated, randomized sequence. The randomized allocation will be concealed in double-enclosed, opaque, sealed, and sequentially numbered envelopes prepared at the University Hospital of Padua.

In the delivery room or operating room, the next sequential randomization envelope will be opened only when the infant will be considered to be eligible by the attending operator. The assigned procedure (PPV with LMA or FM) will then be performed. Multiple births will be separately randomized.

### Blinding

Due to the characteristics of the intervention, neither caregivers nor outcome assessors will be masked to treatment allocation. To minimize bias, strict criteria and definitions will be maintained during the trial.

### Guidelines for management

Before starting the study, all those involved in the neonatal resuscitation participated in a one-day theoretical and practical (manikin) course based on the Neonatal Resuscitation Program (NRP). During the course, one section was dedicated to the preparation and insertion of the size 1 Supreme LMA™ [18]. In November 2011, three courses were held by three certificated NRP teachers in collaboration with an expert in the LMA use (DT). A total of 44 participants (15 physicians and 29 nurses) were trained.

After the course, a period of 3 months was left to routinely introduce LMA use in the delivery rooms. Five successful LMA insertions in the manikin and three LMA insertions in the clinical setting were required to all participants before starting the study.

The American Heart Association and American Academy of Pediatrics Guidelines for Neonatal Resuscitation will be followed in this study [1,2]. After initial steps (warming, clearing airway, drying, stimulation), PPV with FM or LMA and bag will be initiated in the case of apnea and/or gasping and/or heart rate < 100 bpm. The neonate’s trachea will be intubated if the heart rate does not rise or remains less than 60 bpm after 30 seconds of PPV with the LMA or FM. A maximum of three attempts for obtaining effective PPV with an LMA or an FM will be allowed.

Manual ventilation will be initiated in room air at a frequency of 40 to 60 breaths per minute [1,2]. The FiO_2_ will be increased to 1,0 (flow rate 6 to 8 L/min) in case of persistent cyanosis and/or heart rate <100 bpm after 90 seconds from the beginning of PPV. At least two trained people involved in the study will take part in the resuscitation of all enrolled patients.

Resuscitation will start immediately after delivery of the infant, when a stop watch will be switched on by one of the members of the resuscitation team.

The duration of resuscitation will be defined as the time period from starting resuscitation to the establishment of a spontaneous and sustained respiratory pattern of efficacious respiratory movements, which allowed the neonate to maintain adequate clinical parameters (heart and respiratory rate).

In this study, the last model of size 1 LMA Supreme (LMA Supreme™, LMA Company, UK) will be used [18]. Previous studies conducted in adult patients showed the efficacy and the safety of the LMA Supreme [19,20]. The LMA Supreme is superior to the LMA Classic™ with regard to insertion time and oropharyngeal seal pressure [21]; a further advantage consists in the gastric access.

A previous neonatal manikin study confirmed these findings, including a higher level of satisfaction expressed by users [22].

### Data collection

Data will be recorded from clinical records and from a data sheet designed for this study, where all the data obtained during resuscitation procedures will be collected by an observer not involved in the resuscitation maneuvers. Registered clinical information will be: eligibility, antenatal history, randomization, and all data above listed in the ‘Primary outcome measure’, ‘Secondary outcome measures’ and ‘Other collected data’ sections. Further information will be collected on expected serious adverse events (SAEs).

### Statistical analysis

Categorical data will be expressed as number and percentage and compared using Fisher’s test. Continuous data will be expressed as mean and standard deviation or median and interquartile range. The normality assumption of continuous variables will be evaluated using Shapiro-Wilk test. Continuous data will be compared using Student’s *t*-test or the Mann-Whitney non-parametric test. Correlation between continuous data will be evaluated using the Pearson correlation coefficient or the Spearman correlation coefficient. A *P*-value less than 0.05 will be considered significant. Statistical analysis will be performed using the R 2.12 language [23].

### Duration of study

In this study, 142 infants will be recruited. The trial will terminate when the last recruited infant is discharged from hospital, or dies.

### Ethics committee approval

The C hospital, Hanoi, Vietnam Ethis Committees for Human Investigation approved the study (SO:901/QD-PSTW; Ha Noi, 9 August 2012).

### Compliance to protocol

Compliance will be defined as full adherence to protocol. Compliance with the protocol will be ensured by two members of the project (TDC, NTTH) responsible for local data collection. They will weekly monitor the adherence to the study protocol and will input the patients’ data in an Excel data sheet.

### Safety

Safety measures will include incidence, severity, and causality of reported SAEs, represented by changes in occurrence of the expected common neonatal complications and the development of unexpected SAEs. All SAEs will be followed until complete resolution or until the clinician responsible for the care of the recruited patient considers the event to be chronic or the infant to be stable. If there is a reasonable suspected causal relationship with the intervention, SAEs will be reported to the Ethics Committee to guarantee the safety of the participants.

## Discussion

There are unique features of this trial compared to prior studies on the use of the LMA during neonatal resuscitation. To our knowledge, three randomized controlled trials including 140 patients have been previously published [12]. Due to the limited number of enrolled patients, a final conclusion cannot be drawn. A further trial with a different study design (quasi- randomized controlled trial) showed that the LMA significantly reduced the need of intubation in the delivery room in comparison with FM ventilation [17]. All these studies were performed with a classic LMA. In this trial, we used, for the first time, a more advanced model of LMA, the LMA Supreme.

## Trial status

The trial is currently recruiting study subjects.

## Abbreviations

BPM: beats per minute; ETT: endotracheal tube; FM: face mask; HIE: hypoxic-ischemic encephalopathy; LMA: laryngeal mask airway; NRP: Neonatal Resuscitation Program; PPV: positive pressure ventilation; SAEs: serious adverse events; SLMA: supreme laryngeal mask airway.

## Competing interests

The authors declare that they have no competing interests.

## Authors’ contributions

DT, FC, VM, NNL, NVT, TDL, TDC, ND, LC, and LM have made substantial contributions to the conception and design of the study protocol, and have given final approval of the version to be published. All authors read and approved the final manuscript.
